# Distal femur fractures: The use of a fibular strut allograft with dual locking plates allows for early weight bearing*

**DOI:** 10.37796/2211-8039.1642

**Published:** 2025-03-01

**Authors:** Brian R.K. Chee, Chenghan Wu, Abhjeet A. Salunke, Yongsheng Chen

**Affiliations:** a Department of Orthopaedic Surgery, Ng Teng Fong General Hospital, Singapore; b National Healthcare Group, Singapore; c The Gujarat Cancer and Research Institute (GCRI), Ahmedabad, Gujarat, India

**Keywords:** Distal femur, Early weight bearing, Fibular allograft

## Abstract

**Introduction:**

Distal femur fractures result in high morbidity and mortality - comparable to that of hip fractures. The commonly used surgical fixation techniques today, locked plating and intramedullary nailing, have shown high postoperative complication rates. Thus, many surgeons temporarily keep patients non-weight bearing in the early postoperative stage. Increased time to ambulation after surgery is known to increase systemic complications in patients. We aim to investigate if an augmented fixation technique involving the use of a fibular strut allograft with dual locking plates helps to allow early mobilization postoperatively without adverse outcomes.

**Methods:**

Five geriatric patients (four female, one male) with distal femur fractures (native or periprosthetic) were treated in our institution with the aforementioned technique, and were allowed early postoperative weight-bearing. These patients were followed up for postoperative outcomes. The primary outcomes studied were non union, implant failure and wound complications. Secondary outcomes studied include time to union, and Sander’s functional score.

**Results:**

There were no cases of non-union, implant failure or wound related infection. All patients achieved radiological union (mean = 12.6 weeks). Using Sander’s functional scoring, two patients achieved excellent, two achieved good and one had fair outcomes. All patients were followed up for at least 6 months after operation.

**Conclusion:**

Our method of augmented fixation with fibular strut allografts potentially allows for early weight bearing without adverse outcomes. Further studies with larger sample sizes are required to validate our findings.

## 1. Introduction

Fractures of the distal femur represent 0.5 % of all fractures, and 4–6 % of femoral fragility fractures [1].

While uncommon, they can result in high morbidity and mortality in the elderly, similar to hip fractures [2].

These fractures most commonly affect the elderly, female population [3–6]. In some studies, up to 83 % of patients were female [1,3], and the mean age at time of fracture above 60 years old (62.2–67.3) [1,3].

Another significant subset of distal femur fractures are periprosthetic. In a recent study examining 302 distal femur fractures, 27.8 % were periprosthetic [3].

With the world experiencing an aging population, and incidence of total knee arthroplasty (TKA) procedures rising, we can expect distal femur fractures, both native and periprosthetic, to become increasingly common.

A wide variety of surgical options are available in management of distal femur fractures - however, no consensus has been reached on which option is best. Two popular options, plating and intramedullary nailing [7,8] have shown similar clinical outcomes in recent retrospective studies and systematic reviews [9–12]. There remains a need for further research to determine the “gold standard” with regards to treatment of distal femur fractures [13].

High postoperative complication rates, both local and systemic, continue to plague surgical fixation of distal femur fractures. In a study of over 500 distal femur fractures, revision rates of distal femur fractures fixed with plates was 13.6 % [14]. Non-union was the most common complication leading to revision. Poor quality of bone, as well as inadequate stabilization have been identified as some of the factors contributing to non union [15,16].

Due to concerns of implant failure, many surgeons choose to restrict weight bearing in the early postoperative stages [17]. This prolonged immobility can contribute to high rates of systemic complications such as urinary tract infections, DVT/PE and pneumonia - up to 38 % in some studies [18].

Some surgeons have begun to explore new techniques, such as nail-plate constructs, to achieve more stable fixation, aiming to reduce implant failure rates and allow early weight bearing [19,20].

Levack et al. describes the use of plate augmentation with fibular strut allografts in periprosthetic distal femur fractures, providing additional stability and allowing for better cortical screw fixation [21].

However, to the best of our knowledge, there is no consensus on the optimal surgical technique to allow early mobilization and safe weight bearing in these geriatric patients.

We seek to highlight, in our series of patients, that the use of fibular strut allografts together with dual locking plates in distal femur fractures helps to allow early mobilization postoperatively - with good functional outcomes and low complication rates.

## 2. Methods

Upon approval by the Institutional Review board, we conducted a study examining geriatric patients who were treated for distal femur fractures in Ng Teng Fong General Hospital (Singapore), between January 2019 and December 2023.

The inclusion criteria were patients over the age of 65 with isolated distal femur fractures, either native or periprosthetic, who had undergone surgery involving dual plating of the fracture with the use of a fibular strut allograft.

The exclusion criteria included polytrauma patients, those with pathological fractures and patients who were deemed not fit for surgery.

Five patients (four female, one male), comprising four native and one periprosthetic distal femur fractures, were included in our study. The medical records and radiographs of these patients were retrospectively accessed.

These selected patients were included in our study as they fulfilled the following criteria: firstly, these patients sustained isolated injuries with no other concomitant injuries - as other injuries may preclude early weight bearing, or may interfere in post operative rehabilitation. Secondly, these patients were chosen as they had relatively good ambulatory status prior to injury - all except one were ambulatory in the community, with the remaining patient being able to ambulate independently at home. Thus, these patients would be suitable candidates for weight bearing during post operative rehabilitation, and this also gives us a good baseline to compare to when determining functional outcomes. Thirdly, these patients were selected as their radiographs showed significant osteopenia, and fracture patterns with metaphyseal comminution - these types of fractures would benefit most from the additional mechanical benefits of our proposed technique.

Electronic medical records were accessed for data collection. The primary outcomes studied were rates of non union, implant failure and wound complications, while secondary outcomes studied include time to union, and Sander’s functional score.

## 3. Surgical technique

Under general anesthesia, the procedure is performed with the patient in a supine position.

The surgical approach involves a modified anterior approach to the knee. A midline incision is performed with a lateral parapatellar approach, allowing direct visualization of the articular surface along with the fracture site. A swashbuckler extension is then performed to allow the application of dual locking plates.

After the fracture is adequately exposed, the fibula allograft is inserted as a strut graft in a retrograde manner through the fracture site.

Anatomical reduction of the fracture site is then provisionally achieved with wiring.

Aided by an image intensifier, reduction and fixation of the lateral column is performed with a locking compression plate (LCP), maximizing bony purchase with up to six locking screws. Medial column fixation is then completed with another LCP. The screws are planned and placed in a manner where there is interdigitation with the fibula strut allograft.

The wound is then closed in a routine manner with repair of the arthrotomy.

After review of post operative plain radiographs, post-op rehabilitation is started from post-operative day 1. The patient is allowed weight bearing and ranging of the left lower limb as tolerated, with the assistance of a physical therapist and with appropriate walking aids.

Antibiotic prophylaxis is given prior to operation and continued for 24 h post operatively.

Clinical and radiographic evaluations were performed at 6 weeks, 3 months, 6 months and 1 year after surgery, with subsequent follow ups being determined by the performing surgeon.

## 4. Case report

### 4.1. Native distal femur fracture

**Fig. 1:**
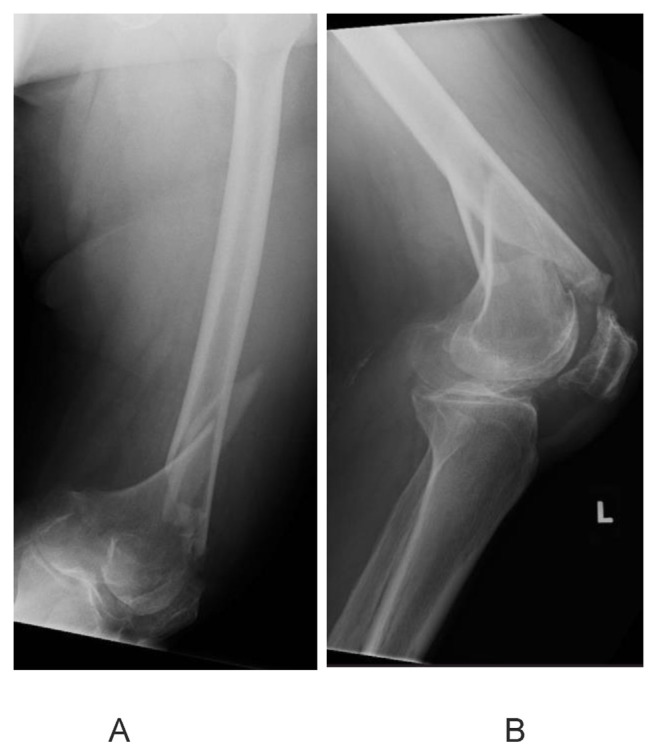
Preoperative radiographs show a comminuted, extra-articular fracture of the left distal femur.

**Fig. 2:**
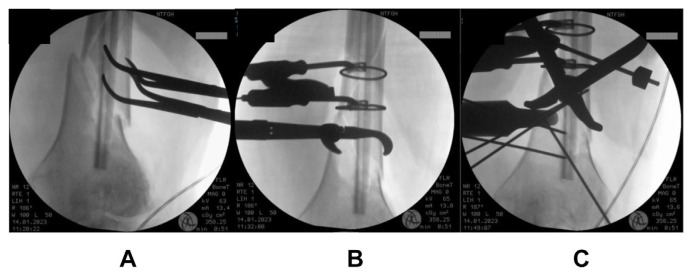
Intraoperative radiographs show the fibular strut allograft placed within the fracture site, before reduction and temporary fixation with K wires.

**Fig. 3:**
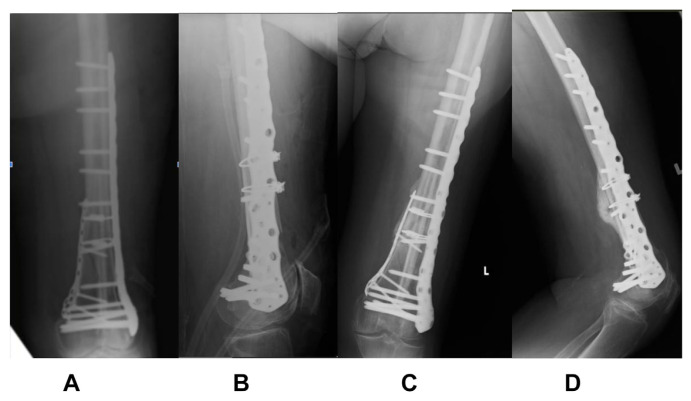
(A and B) Immediate postoperative radiographs. (C and D) Radiographs at 6 months after surgery.

**Fig. 4:**
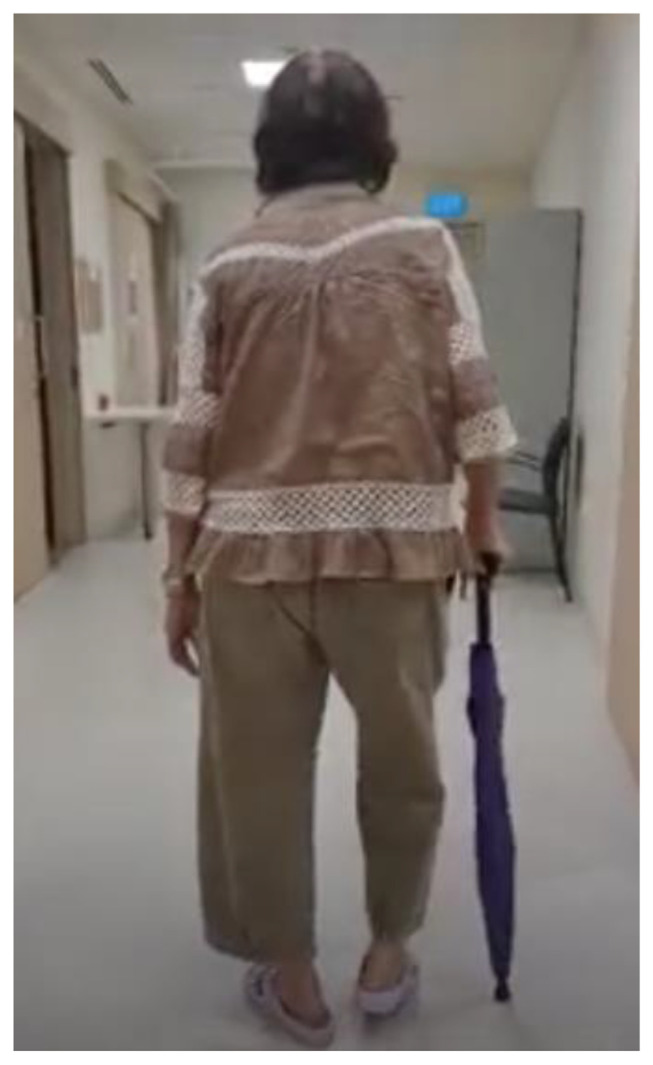
The patient is shown ambulating well with a walking aid at 6 months after surgery.

A 73-year old lady, with a significant medical history of hypertension and diabetes mellitus, sustained a left knee injury after a fall. Imaging revealed a comminuted, extra-articular fracture of the left distal femur (Fig. 1 A and B). The patient subsequently underwent surgical fixation. Intraoperative radiographs (Fig. 2 A–C) show the fibular strut allograft being placed within the fracture site, before reduction and temporary fixation with K wires. Additional cerclage wiring was used to strengthen the construct in view of proximal extension of the fracture line. Immediate postoperative radiographs (Fig. 3 A and B) as well as at 6 months (Fig. 3 C and D) show the final construct comprising two LCPs, multiple cortical screws, cerclage wiring and the fibular allograft in-situ. The patient was allowed weight bearing as tolerated after the operation. At 6 months of follow up, the patient was ambulating well with a walking aid (Fig. 4).

### 4.2. Periprosthetic distal femur fracture

**Fig. 5:**
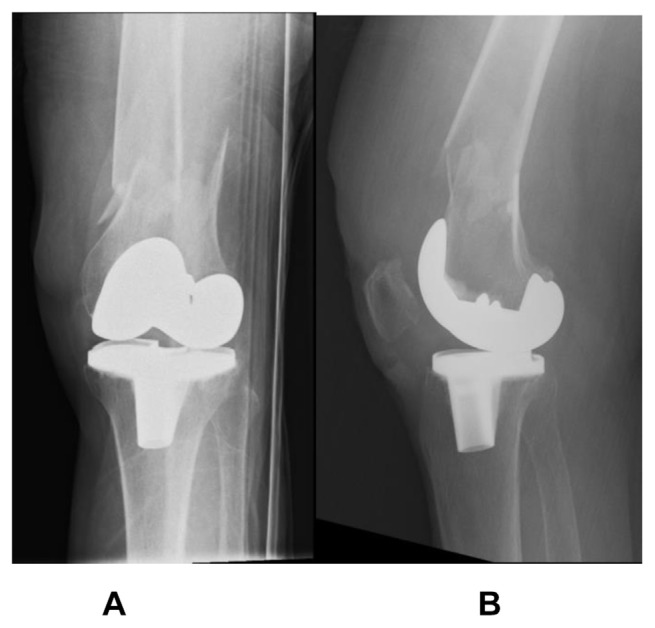
Preoperative radiographs show a periprosthetic fracture of the right distal femur.

**Fig. 6:**
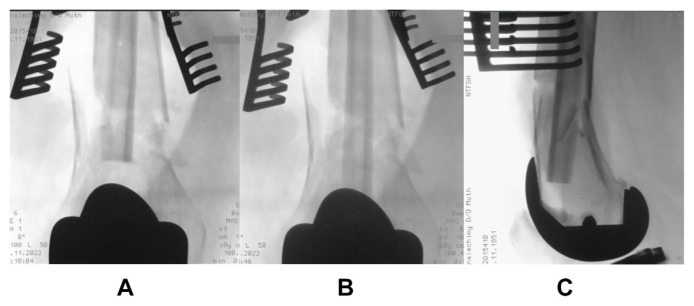
Intraoperative radiographs show the fibular strut allograft being inserted through the fracture site and distalized, helping to maintain length.

**Fig. 7:**
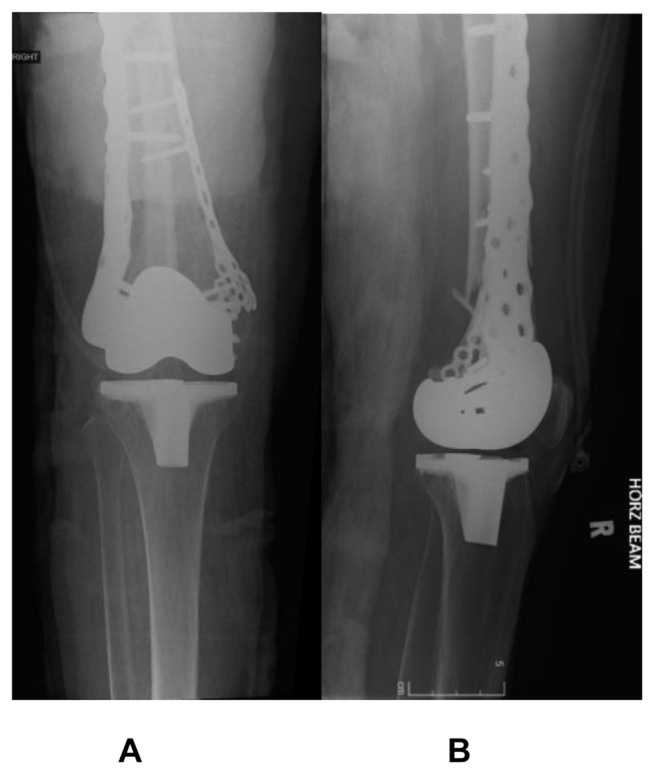
Immediate postoperative radriographs.

**Fig. 8:**
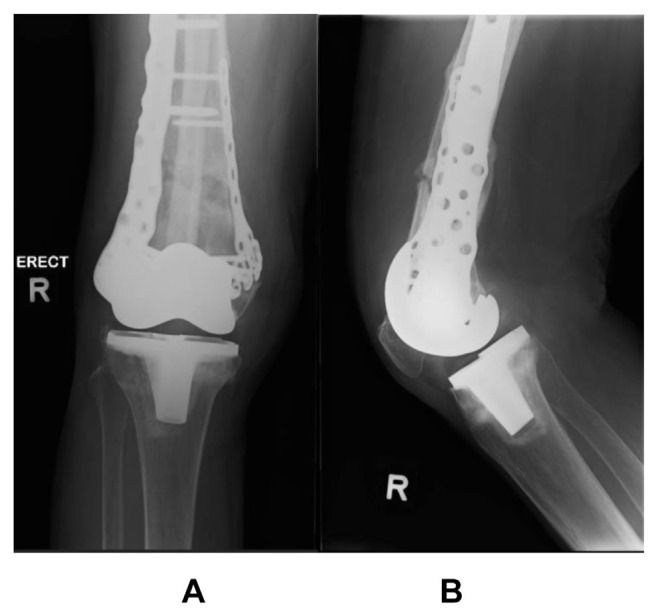
Radiographs at 6 months after surgery.

A 71-year old lady, with previous bilateral total knee replacements, was involved in a road traffic accident. She suffered a periprosthetic fracture of her right distal femur (Fig. 5 A and B), with significant metaphyseal comminution and shortening. Intra-operatively, the fibular strut allograft is inserted via the fracture site and distalized, helping to maintain length (Fig. 6 A–C). The immediate (Fig. 7 A and B) and 6-months radiographs (Fig. 8) are shown. The patient was able to ambulate pain-free with a walking aid at 6 months.

## 5. Patient characteristics and results

Five patients, four female and one male, were treated with the aforementioned technique, with a mean age of 73.2 years. Patients were followed up for at least 6 months.

All patients achieved fracture union. The average time to radiographic union was 12.6 weeks. At the time of last follow up, there were no incidences of systemic complications, wound infection or implant failure. Using the Sander’s functional score, a scoring system comprising both subjective and objective markers of function [22], patients’ functional outcomes were grouped into excellent (36–40 points), good (26–35 points), fair (16–25 points) or poor (0–15 points). Two patients achieved excellent, two patients achieved good while one had a fair outcome.[Table t1-bmed-15-01-057]

**Table t1-bmed-15-01-057:** 

No.	Age	Sex	Side	Comorbidities	Pre-morbid ambulatory status	Periprosthetic?	Fracture Classification	Follow up period	Complications	Time to union, in weeks	Sander’s functional score at last follow up
1	73	F	L	Hypertension, diabetes mellitus	Comm amb with walking aid	No	AO/OTA 33A2	7mo	Nil	12	Excellent
2	76	M	L	Hypertension, gout	Homebound independent	No	AO/OTA 33B2	6mo	Nil	15	Fair
3	81	F	L	Hypertension, right total knee replacement	Comm amb with walking aid	No	AO/OTA 33C2	9mo	Nil	12	Excellent
4	71	F	R	Hypertension, diabetes mellitus, bilateral total knee replacement	Comm amb without aid	Yes	Rorabeck type II	6mo	Nil	13	Good
5	65	F	L	Hypertension, diabetes mellitus	Comm amb without aid	No	AO/OTA 33-C3	6mo	Nil	11	Good

## 6. Discussion

The results of our study suggest that our method of augmented fixation with fibular strut allografts for distal femur fractures in geriatric patients safely allows for early weight bearing in patients, and achieves good clinical outcomes with low postoperative complications.

Distal femur fractures occur most commonly in the geriatric population, and result in significant morbidity and mortality, similar to that of hip fractures.

Unlike hip fractures, for which several published guidelines by various organizations (e.g the American Academy of Orthopaedic Surgeons) are available and well established, there is ongoing debate regarding the standard of care for distal femur fractures [23].

Today, it is generally accepted that surgical fixation results in better outcomes than conservative management, including improved functional outcomes and union rates, and reduced complication rates [24,25].

Despite this, when compared to other fracture types, locked plating and intramedullary nailing of distal femur fractures continue to show high rates of complications such as implant failure and non union. This can be attributed to various factors, including poor bone quality in elderly patients, significant metaphyseal comminution seen in these fractures, and inadequate stabilization of the nail or plate construct.

Vemulapalli et al. compared locked lateral plating and retrograde nailing for distal femur fractures, reporting nonunion rates of 11.8 % in the nailing group and 27.5 % in the plating group - these numbers are on the higher end of nonunion rates quoted in the current literature.

In a recent systematic review and meta-analysis comparing locked plating and intramedullary nailing conducted by Neradi et al., non union rates were 7.8 % in the nailing group and 11.4 % in the plating group [26]. Implant failure was exclusively seen in the plating group - with a rate of 8 %. This may be explained by the intrinsic nature of an intra-medullary nail being a load sharing device, compared to that of a plate which bears the full functional load.

Similar numbers were reported by Collinge et al. who analyzed over 1000 distal femur fractures treated with distal femoral locking plates. They reported an implant failure rate of 9.3 %, and non union rate of 13.4 % [27].

On the other hand, separate systematic reviews and meta-analyses on studies comparing the previously mentioned fixation techniques by Shah et al. and Yoon et al. showed more conservative complication rates. Shah et al. reported non union rates of 6.4 % and 8.3 %, and implant failure rates of 5.0 and 6.3 %, while Yoon et al. reported non union rates of 4 % and 6 % [12,31].

Recently, a number of studies and a randomized control trial comparing postoperative early weight bearing versus non weight bearing in distal femur fractures after locked plate fixation have shown similar non union and implant failure rates in the two groups [28,29]. Despite this, it remains common practice amongst surgeons to restrict weight bearing in the postoperative period due to concerns of the aforementioned complications [17].

Several alternative surgical techniques have been explored in view of the existing problems associated with the commonly used fixation techniques.

A combined nail-plate construct has been proposed by various authors to provide greater stability with promising outcomes [19,20].

More recently, some surgeons have begun exploring the use of a fibular strut graft as a “biological nail” to augment plating in distal femur fractures. This has been described in the following two studies.

Levack et al.’s study describes the use of fibular allograft with dual plating in periprosthetic distal femur fractures with good early postoperative outcomes [21]. Ibrahim et al.’s retrospective case series of 30 distal femur fracture patients treated with primary fibular grafting combined with double plating showed higher rates of union and lower revision rates than other fixation modalities [30]. In the latter study, patients were allowed full weight bearing at 3 months post-operation.

Our study supports the above studies’ findings that this method of augmented fixation is an effective means of fixation in distal femur fractures, and can be applied to both the native and periprosthetic knee. In addition, our study also shows that our proposed technique allows safe and early mobilization in geriatric patients after fixation, without compromising on implant failure rates.

Distal femur fractures are known to be challenging to manage as they tend to produce significant metaphyseal comminution. This, combined with osteoporotic bone in the elderly population, leads to bone loss and the formation of a metaphyseal gap after fracture reduction - contributing to high postoperative complication rates.

Our proposed technique has multiple benefits in overcoming these challenges - firstly, the fibular strut graft helps to fill this metaphyseal void that is created. Secondly, it increases the purchase of the cortical screws in osteoporotic bone which helps to reduce the risk of screw back-out. The fibular strut graft also aids in maintaining reduction and retaining femur length intraoperatively before definitive fixation is attained. Allografting also has the advantage of reduced operating time - as time would be saved harvesting the autograft. Overall, augmented fixation with a fibular strut allograft leads to increased stability of implants across the fracture site.

However, we recognise that the use of an allograft adds additional cost to the surgery. In areas that are less resource rich, the cost and procurement of allografts may be a limiting factor in adoption of our proposed technique.

To the best of our knowledge, no previous studies have discussed the use of fibular strut allografting with dual plating in both native and periprosthetic distal femoral fractures. Furthermore, there is also a limited number of studies discussing the outcomes of early weight bearing postoperatively in distal femur fractures. We hope to use our series of patients to demonstrate the effectiveness of our proposed technique in helping geriatric patients mobilize earlier after surgery for distal femur fractures.

We postulate that this will help to reduce mortality and morbidity in this population, as prolonged immobility is associated with a host of systemic complications. We have identified a few shortcomings in our study. Firstly, our case series contains a small sample of patients - further studies with larger sample sizes would help to validate our findings. Furthermore, the patient profile of our limited number of patients are quite similar. Most of our patients are geriatric, female, community ambulant and with the absence of pre-morbid debilitating medical conditions. Although our patient sample mirrors the demographics of a significant number of distal femur fractures (elderly, female), our results may not be generalizable to the other spectrum of patients who are male, younger, or patients who are more frail and may not be sufficiently robust for early mobilization.

Secondly, our patients had a follow up period of 6 months. While this is adequate to study union rates and early implant failure, this may prevent us from observing longer term complications such as late implant loosening and mortality and morbidity in the long run.

In conclusion, our technique of using a fibular strut allograft to augment dual locking plates in osteoporotic distal femur fractures allows for early weight bearing safely, produces good functional outcomes, and does not require subsequent surgery for revision surgery within the observed period of follow-up. We propose that this is a replicable technique that can be used to achieve good results in the geriatric population.

## References

[1] Court-Brown CM Heckman JD McQueen MM Ricci WM Tornetta P McKee MD Rockwood and Green’s Fractures in Adults eighth ed Philadelphia Wolters Kluwer Health 2015

[2] Loosen A Fritz Y Dietrich M Surgical treatment of distal femur fractures in geriatric patients Geriatr Orthop Surg Rehabil 2019 10 2151459319860723 31308992 10.1177/2151459319860723 PMC6607559

[3] Elsoe R Ceccotti AA Larsen P Population-based epidemiology and incidence of distal femur fractures Int Orthop 2018 42 191 6 29116356 10.1007/s00264-017-3665-1

[4] Ng AC Drake MT Clarke BL Sems SA Atkinson EJ Achenbach SJ Trends in subtrochanteric, diaphyseal, and distal femur fractures, 1984–2007 Osteoporos Int 2012 23 1721 6 21909727 10.1007/s00198-011-1777-9 PMC3266989

[5] Kolmert L Wulff K Epidemiology and treatment of distal femoral fractures in adults Acta Orthop Scand 1982 53 957 62 7180408 10.3109/17453678208992855

[6] Court-Brown CM Caesar B Epidemiology of adult fractures: a review Injury 2006 37 691 7 16814787 10.1016/j.injury.2006.04.130

[7] Hake ME Davis ME Perdue AM Goulet JA Modern implant options for the treatment of distal femur fractures J Am Acad Orthop Surg 2019 27 e867 75 30939565 10.5435/JAAOS-D-17-00706

[8] Gangavalli AK Nwachuku CO Management of distal femur fractures in adults: an overview of options Orthop Clin N Am 2016 47 85 96 10.1016/j.ocl.2015.08.011 26614924

[9] Hoskins W Sheehy R Edwards ER Hau RC Bucknill A Parsons N Nails or plates for fracture of the distal femur? Data from the victoria orthopaedic trauma outcomes registry Bone Joint Lett J 2016 98B 846 50 10.1302/0301-620X.98B6.36826 27235531

[10] Park J Lee JH Comparison of retrograde nailing and minimally invasive plating for treatment of periprosthetic supracondylar femur fractures (OTA 33-A) above total knee arthroplasty Arch Orthop Trauma Surg 2016 136 331 8 26646847 10.1007/s00402-015-2374-8

[11] Ebraheim NA Kelley LH Liu X Thomas IS Steiner RB Liu J Periprosthetic distal femur fracture after total knee arthroplasty: a systematic review Orthop Surg 2015 7 297 305 26790831 10.1111/os.12199 PMC6583744

[12] Shah JK Szukics P Gianakos AL Liporace FA Yoon RS Equivalent union rates between intramedullary nail and locked plate fixation for distal femur periprosthetic fractures - a systematic review Injury 2020 51 1062 8 32115204 10.1016/j.injury.2020.02.043

[13] Smith JR Halliday R Aquilina AL Morrison RJ Yip GC McArthur J Distal femoral fractures: the need to review the standard of care Injury 2015 46 1084 8 25840789 10.1016/j.injury.2015.02.016

[14] Koso RE Terhoeve C Steen RG Zura R Healing, nonunion, and re-operation after internal fixation of diaphyseal and distal femoral fractures: a systematic review and meta-analysis Int Orthop 2018 42 2675 83 29516238 10.1007/s00264-018-3864-4

[15] Ebraheim NA Martin A Sochacki KR Liu J Nonunion of distal femoral fractures: a systematic review Orthop Surg 2013 5 46 50 23420747 10.1111/os.12017 PMC6583155

[16] Ricci WM Streubel PN Morshed S Collinge CA Nork SE Gardner MJ Risk factors for failure of locked plate fixation of distal femur fractures: an analysis of 335 cases J Orthop Trauma 2014 28 83 9 23760176 10.1097/BOT.0b013e31829e6dd0

[17] Consigliere P Iliopoulos E Ads T Trompeter A Early versus delayed weight bearing after surgical fixation of distal femur fractures: a non-randomized comparative study Eur J Orthop Surg Traumatol 2019 29 1789 94 31267203 10.1007/s00590-019-02486-4

[18] Moloney GB Pan T Van Eck CF Patel D Tarkin I Geriatric distal femur fracture: are we underestimating the rate of local and systemic complications? Injury 2016 47 1732 6 27311551 10.1016/j.injury.2016.05.024

[19] Passias BJ Emmer TC Sullivan BD Gupta A Myers D Skura BW Treatment of distal femur fractures with a combined nail-plate construct: techniques and outcomes J Long Term Eff Med Implants 2021 31 15 26 10.1615/JLongTermEffMedImplants.2021038016 34369718

[20] Liporace FA Yoon RS Nail Plate combination technique for native and periprosthetic distal femur fractures J Orthop Trauma 2019 33 e64 8 30277982 10.1097/BOT.0000000000001332

[21] Levack AE Gadinsky N Gausden EB Klinger C Helfet DL Lorich DG The use of fibular allograft in complex periarticular fractures around the knee Operat Tech Orthop 2018 28 141 51 10.1053/j.oto.2018.07.004 PMC6405238 30853772

[22] Sanders R Swiontkowski M Rosen H Helfet D Double-plating of comminuted, unstable fractures of the distal part of the femur J Bone Joint Surg Am 1991 73 341 6 2002071

[23] Bhandari M Swiontkowski M Management of acute hip fracture N Engl J Med 2017 377 205362 10.1056/NEJMcp1611090 29166235

[24] Healy WL Brooker AF Jr Distal femoral fractures. Comparison of open and closed methods of treatment Clin Orthop Relat Res 1983 174 166 71 6831801

[25] Butt MS Krikler SJ Ali MS Displaced fractures of the distal femur in elderly patients. Operative versus non-operative treatment J Bone Joint Surg Br 1996 78 110 4 8898139

[26] Neradi D Sodavarapu P Jindal K Kumar D Kumar V Goni V Locked plating versus retrograde intramedullary nailing for distal femur fractures: a systematic review and meta-analysis Arch Bone Jt Surg 2022 10 141 52 35655740 10.22038/abjs.2021.53515.2656 PMC9117898

[27] Collinge CA Reeb AF Rodriguez-Buitrago AF Archdeacon MT Beltran MJ Gardner MJ Analysis of 101 mechanical failures in distal femur fractures treated with 3 generations of precontoured locking plates J Orthop Trauma 2023 37 8 13 35862769 10.1097/BOT.0000000000002460

[28] Paulsson M Ekholm C Jonsson E Geijer M Rolfson O Immediate full weight-bearing versus partial weight-bearing after plate fixation of distal femur fractures in elderly patients. A randomized controlled trial Geriatr Orthop Surg Rehabil 2021 12 21514593211055889 35145761 10.1177/21514593211055889 PMC8822340

[29] Keenan OJF Ross LA Magill M Moran M Scott CEH Immediate weight-bearing is safe following lateral locked plate fixation of periprosthetic distal femoral fractures Knee Surg Relat Res 2021 33 19 34172101 10.1186/s43019-021-00097-0 PMC8229296

[30] Ibrahim FM Ghazawy AKE Hussien MA Primary fibular grafting combined with double plating in distal femur fractures in elderly patients Int Orthop 2022 46 2145 52 35579697 10.1007/s00264-022-05441-x PMC9371996

[31] Yoon BH Park IK Kim Y Oh HK Choo SK Sung YB Incidence of nonunion after surgery of distal femoral fractures using contemporary fixation device: a meta-analysis Arch Orthop Trauma Surg 2021 141 225 33 32388648 10.1007/s00402-020-03463-x

